# Enabling Model-Based Design for Real-Time Spike Detection

**DOI:** 10.1109/OJEMB.2025.3537768

**Published:** 2025-02-03

**Authors:** Mattia Di Florio, Yannick Bornat, Marta Carè, Vinicius Rosa Cota, Stefano Buccelli, Michela Chiappalone

**Affiliations:** Department of Informatics, Bioengineering, Robotics, System Engineering (DIBRIS)University of Genova 361527 16145 Genova Italy; Laboratoire de l'Intégration du Matériau au Système (IMS)University of Bordeaux, Bordeaux INP, CNRS UMR 5218 27086 33405 Talence Cedex France; IRCCS Ospedale Policlinico San Martino 9246 16132 Genova Italy; Rehab Technologies LabIstituto Italiano di Tecnologia 121451 16163 Genova Italy; Department of Informatics, Bioengineering, Robotics, System Engineering (DIBRIS)University of Genova 361527 16145 Genova Italy; IRCCS Ospedale Policlinico San Martino 9246 16132 Genova Italy

**Keywords:** Signal processing, in vivo experiments, HDL coder, Field-Programmable Gate Array (FPGA), neuroengineering

## Abstract

*Goal*: This study addresses the inherent difficulties in the creation of neuroengineering devices for real-time neural signal processing, a task typically characterized by intricate and technically demanding processes. Beneath the substantial hardware advancements in neurotechnology, there is often rather complex low-level code that poses challenges in terms of development, documentation, and long-term maintenance. *Methods*: We adopted an alternative strategy centered on Model-Based Design (MBD) to simplify the creation of neuroengineering systems and reduce the entry barriers. MBD offers distinct advantages by streamlining the design workflow, from modelling to implementation, thus facilitating the development of intricate systems. A spike detection algorithm has been implemented on a commercially available system based on a Field-Programmable Gate Array (FPGA) that combines neural probe electronics with configurable integrated circuit. The entire process of data handling and data processing was performed within the Simulink environment, with subsequent generation of hardware description language (HDL) code tailored to the FPGA hardware. *Results*: The validation was conducted through in vivo experiments involving six animals and demonstrated the capability of our MBD-based real time processing (latency <= 100.37 µs) to achieve the same performances of offline spike detection. *Conclusions*: This methodology can have a significant impact in the development of neuroengineering systems by speeding up the prototyping of various system architectures. We have made all project code files open source, thereby providing free access to fellow scientists interested in the development of neuroengineering systems.

## I. Introduction

Due to significant investments made by research institutions and medical companies in advancing neurotechnologies for brain disorder treatments [1], contemporary data acquisition techniques now enable the simultaneous recording from thousands of electrodes [2]. Notably, recently developed invasive recording systems offer exceptional spatial and temporal resolution, even down to the single-cell level [3], [4], thus expanding the horizons of in-vivo research. These technological enhancements have spurred substantial advancements in the fields of bioelectronics and neural engineering. Consequently, they have paved the way for the creation of cutting-edge Brain Computer Interfaces (referred to as BCI for simplicity), neuroprosthetic devices, and innovative 'electroceutical' approaches [5]. Electroceutical is a recently defined application field, which consists of treatments based on ‘electrical’ rather than ‘pharmacological’ therapy. Electrical therapies have sparked interests among researchers, industries and funding agents [6], [7]. Electroceutical approaches have already been successfully employed to neurological diseases, such as depression, epilepsy, gastroparesis and arrhythmia, as reported in the literature [8], [9].

Electroceutical therapy can be administered in two ways: by means of open-loop stimulation (i.e., OLS) and closed-loop stimulation (i.e., CLS). OLS implies the delivery of a stimulation pattern without considering the brain's current state, whereas CLS allows to modulate the electrical stimuli based on the brain's ongoing activity [10]. Indeed, closed-loop based-systems were proven to be highly advantageous in several aspects, including biological safety and therapeutic efficacy. With the goal of restoring motor function following stroke or traumatic brain injury, Guggenmos and colleagues [11] used a closed-loop approach inspired by Hebbian plasticity mechanisms. In their novel system, electrical stimuli were applied to the primary somatosensory area (S1) in a time-locked synchronous one-to-one manner to the spikes of neurons in the rostral forelimb area (RFA; the pre-motor area equivalent in rats). Termed ADS (Activity Dependent Stimulation), the approach has been successfully used to improve the performance of rats in reaching and grasping tasks by functionally reconnecting the sensorimotor loop.

The exploitation of closed-loop paradigms has led to an increase in demand for high-performance signal processing techniques for hardware implementation to be utilized in both clinical practice and basic research applications. It also implies the development of real-time algorithms to cope with the higher number of recording sites needed to study the complex spatial and temporal dynamics of the nervous system [12], [13].

The realization of those systems is a demanding process which involves three main steps: (i) recording neural activity at various levels of temporal and spatial resolution and risk; (ii) identifying a neural pattern or decoding the neural activity using real-time signal processing algorithms; (iii) generating a sequence of electrical stimuli based on the output of the processing technique. Field-Programmable Gate Arrays (FPGAs) are often the first choice to create highly reconfigurable systems for different purposes while maintaining very high performance. However, the complexity involved in designing an FPGA architecture for a neuroengineering system, particularly the signal processing stage, can be daunting. This requires deep knowledge of hardware description languages (HDL), which becomes verbose and unportable as hardware design architectures complexity grows [14], [15]. To this end, it is necessary to simplify and speed up the design of FPGA architectures for neural signal processing. Raising the level of abstraction in hardware design is necessary to lower the entry barrier for developing real-time processing systems for neuroengineering applications, thus accelerating research progress. Several high-level synthesis tools (HLS) have been developed, usually based on programming languages such as C++. However, performing hardware design via sequential code leads to lose the ability to control hardware constructs and data scheduling [15]. For this reason, we propose Model-Based Design (MBD) as a solution, being a ‘de facto’ standard in hardware design [16].

The primary objective of this work is to address the existing technological gap by introducing an MBD, specifically Simulink, architecture, for accelerating the implementation of neuroengineering systems on FPGAs. An MBD approach, leveraging the Simulink environment, a well-established MBD tool from MathWorks, along with the HDL coder, could be a game-changer in neuroscientific and neuroengineering research. In fact, Simulink enables simulation of system behavior and troubleshooting of models prior to hardware implementation onto FPGA platforms.

Specifically, by following the introduced approach, we focused on the design of a real-time spike detection algorithm. This method is at the same time central to many real time neural signal processing strategies and computationally straightforward, making it an excellent choice for the demonstration of the validity of the MDB approach. Furthermore, this algorithm has been tested on a commercially available system for in vivo experiments, to be further exploited for closed-loop stimulation. Here, a state-of-the-art threshold-basedspike detection algorithm [17], [18] has been implemented on the FPGA board by means of a custom modification of the original code. The testing phase was necessary to evaluate the performance and reliability of the architecture in real-time conditions. By comparing the offline and online implementations of the same spike detection algorithm, we proved the system's ability to accurately detect spikes in real-time.

This work showcases the potential of MBD methodologies in speeding up the development of real-time signal processing tools for neuroengineering.

The full project, including modified Verilog code, Simulink architecture, library, and documentation, is available on GitHub at the following link: https://github.com/MattiaDif/closed-loop-neuroengineering.

## II. Materials and Methods

### A. General Philosophy of the Approach

**Fig. 1.**
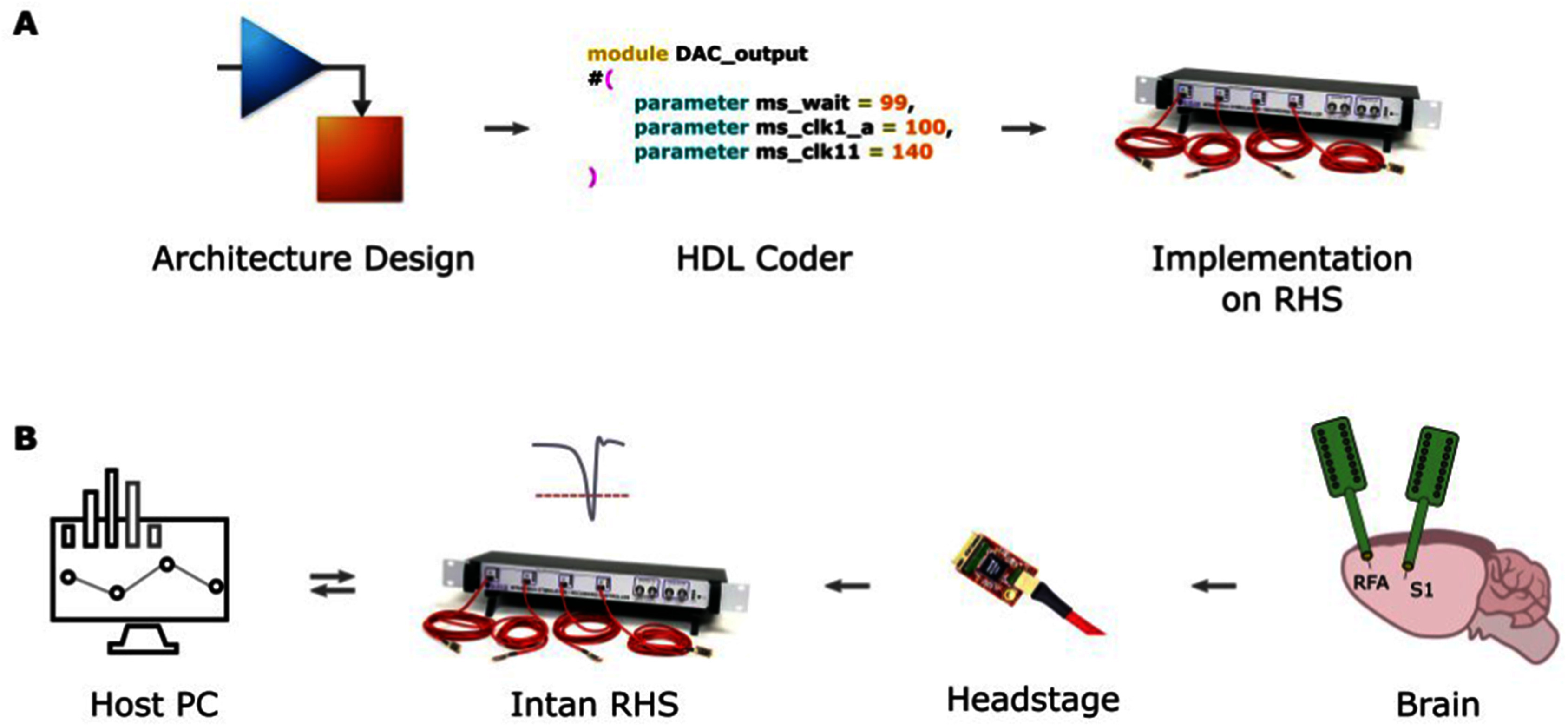
System overview and design steps. A: main steps followed for the model-based design approach. All the architecture has been designed in Simulink, then, exploiting the HDL Coder, Verilog code has been generated and integrated into the original Verilog architecture of the Intan RHS. B: system used for real-time spike detection. A host PC serves as running a Graphical User Interface for online data visualization and control of the Intan RHS parameters. The Intan RHS consists of the core of the system. It is an FPGA-based controller capable of real-time data acquisition and stimulation. By modifying the original code of the controller, a spike detection algorithm has been implemented. The headstage serves as interface between the controller and the electrodes implanted in the animal brain. It amplifies the signal and provides analog to digital conversion.

The proposed MBD approach was achieved through the following steps (see Fig. 1 Panel A): (i) creating a Simulink model for processing neural data. Simulink, being a graphical programming environment, allows for the modeling and simulation of dynamic systems in a faster and more intuitive way with respect to traditional methodologies. By creating a comprehensive Simulink model, the project establishes a foundation for implementing closed-loop systems on FPGAs; (ii) using HDL Coder to implement the model on FPGA. HDL Coder is employed to generate synthesizable Verilog and VHDL code from the Simulink model which can then be programmed onto the FPGA; and (iii) establishing use case for commercially available systems. This use case provides real-world examples of how the architecture can be leveraged in various domains, highlighting its versatility and potential benefits. The Intan Stimulation/Recording system has been selected as the appropriate platform for the study and it is detailed in the following section.

### B. System Overview

The neural signal processing system adopted for the work is the Intan RHS Stim/Recording System. It is an FPGA-based commercially available data acquisition and stimulation system designed for in vivo experiments (see Fig. 1 Panel B) which allows to acquire and deliver intracortical stimulation from up to 128 channels. It consists of three main components: (i) headstage: the headstage incorporates the Intan Technologies RHS2116 microchip. It serves as a bidirectional electrophysiology interface system and facilitates the connection of the microelectrode array. The headstage comprises 16 stimulation/amplifier blocks, which are responsible for amplification and digitization of the neural signals; (ii) acquisition System: the acquisition system is built around the Intan Technologies RHS Stim/Recording controller. This system is FPGA-based and designed specifically for electrophysiology data acquisition. It enables the simultaneous recording of incoming neural signals from the headstage and facilitates communication of stimulation parameters through the interface; and (iii) host PC: the host PC serves as a general-purpose computer that runs software for real-time data visualization and facilitates custom parameter communication. It acts as the central control unit for the overall system. The software running on the host PC provides the necessary tools and interfaces for visualizing the recorded neural data and enables communication with the acquisition system for configuring and controlling stimulation parameters. The host PC acts as an essential interface between the user and the real-time neural signal processing system.

These three components form a full system for real-time neural data acquisition and stimulation. The headstage captures neural signals from the microelectrode array, the acquisition system handles the recording and stimulation parameters, and the host PC provides a user-friendly interface for controlling the FPGA architecture and visualizing the data in real-time.

The Intan RHS can operate at three different clock speeds depending on the set sampling frequency: i) 84 MHz, ii) 70 MHz and iii) 56 MHz for 30 kHz, 25 kHz and 20 kHz of sampling frequencies respectively.

### C. Overall Hardware Architecture

**Fig. 2.**
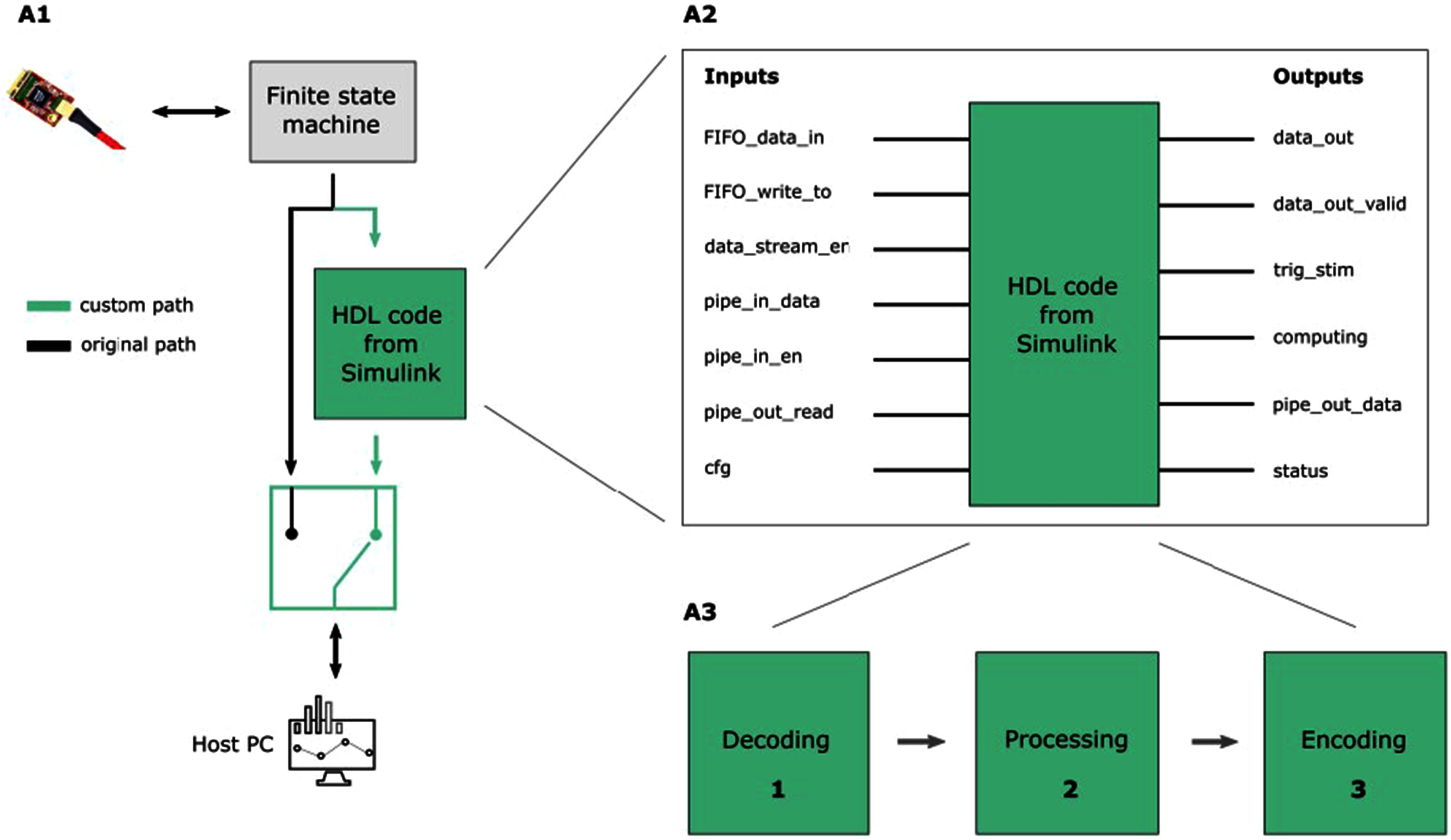
Custom architecture overview. A1: Modification of the original Verilog code of the Intan RHS to redirect the dataflow through the custom path without affecting the initial behavior. The “HDL code from Simulink” block consists in the Verilog code generated from the Simulink model. A2: detail of the I/O interface of the Simulink model. This series of inputs and outputs allow communication between the output of the finite state machine and the custom paths. A3: the HDL block provides custom computation which consists of decoding, processing (filtering and spike detection) and encoding steps.

The Custom Architecture (CA) is a modified version of the original Verilog code (see Fig. 2 Panel A1) provided by the Intan Technologies © company, designed to hold the Verilog code generated by the Simulink model. The CA redirects acquired data to a custom path to process the neural data in real-time. By incorporating a switch in the original path, the dataflow can be diverted through the custom path that houses the custom Verilog code generated from Simulink (see Fig. 2 Panel A1).

The signal processing algorithm of the architecture was developed using the Simulink HDL library provided by MathWorks. After the model design, Verilog code was generated and integrated into the modified Verilog architecture.

The custom computation developed in Simulink consists of three primary steps: data decoding, data processing, and data encoding (see Fig. 2 Panel A3). Data Decoding: this initial step involves the interpretation and conversion of input data into a format that can be readily processed by the subsequent stages. If the input data is in a specific encoding or compressed format, it needs to be decoded and transformed into its original representation. In the custom computation developed in Simulink, the input data is first decoded using a Moore State Machine implemented with StateFlow. The purpose of the Moore State Machine is to determine the channel and headstage from which the input data was acquired. This is required because the acquisition loop returns a complex flow of data which includes information about the headstage register states, sampled data, TTL signals states, etc., and the neural data are acquired serially. It operates by transitioning between different states based on the input data and current state. The process starts once a predefined data is received, a programmed value for synchronization. Therefore, a new acquisition loop begun, and new data will be sampled for all the channels. Starting with this number, the finite-state machine updates its state each time it receives a new input. For specific combinations of current state and input of the state machine, channel number and headstage are identified and sent to the processing module.

**Fig. 3.**
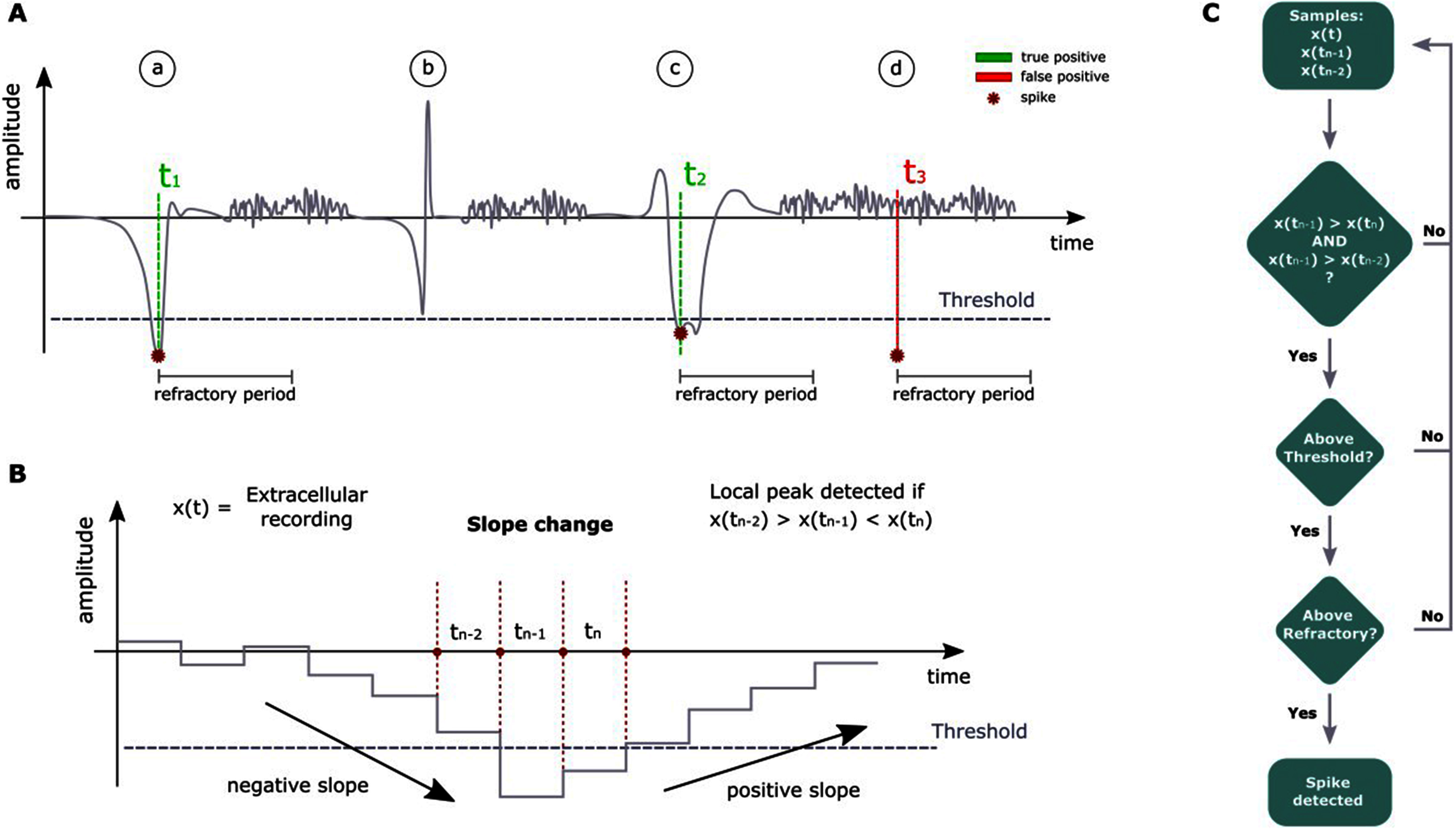
Spike detection algorithm functioning schematic. A: examples of true positive, false negative and false positive detections of the algorithm. (a) An action potential below the threshold is identified correctly (true positive). (b) An action potential above threshold is not identified (false negative). (c) An action potential which shows a double peak is correctly identified just once because of the refractory period that begins as soon as the first peak is detected, avoiding a double detection (true positive). D) A noisy peak below threshold is wrongly detected by the algorithm (false positive). B: Details of algorithm functioning. For the peak detection, the current sample at time t_n_, the previous sample at time t_n-1_ and the sample at time t_n-2_ are considered. In the sample at time t_n-1_ is below threshold and it is the lowest, a spike is identified. C: flow chart to summarize the main steps of the algorithm.

Data Processing: Once the input data is decoded, it undergoes various processing operations to extract relevant information and perform the desired computations. This step typically involves applying algorithms, filters, transformations, or any other necessary operations to manipulate and analyze the data according to the intended purpose. Within the data processing block of the architecture, there are two main steps: filtering the raw data and performing spike detection computations. The first step involves filtering the raw data that is sampled by the headstage. In the current implementation, a 2nd order highpass Butterworth filter is utilized. This filter is designed to remove low-frequency components from the signal, allowing for the preservation of higher-frequency components. The cutoff frequency of the filter is set at 300 Hz. More in detail, the filter consists in a Direct-Form-II architecture, designed from scratch in Simulink. All the samples are processed serially. Intermediate states for that specific channel that is being filtered, are stored in two dual-port RAMs ready to be retrieved during the next acquisition loop. Once the data is filtered, spike detection is performed using a hard threshold spike detection algorithm. In this algorithm, a local peak, specifically a negative one, is identified as an action potential (see Fig. 3). The spike detection is based on a comparison of the current sample at time t, the sample at time t-1, and the sample at time t-2. To identify a spike, two conditions must be met: (i) the sample at time t-1 must be the lowest among the three samples, and (ii) the sample at time t-1 must be lower than a set threshold. If both conditions are satisfied, a spike is identified. Again, considering that all the channels are processed serially, two dual-port RAMs keep track of two previous filtered samples. Once a spike is identified, a refractory period counter begins. This counter is incremented every time a new acquisition loop begins (i.e., a “magic number” is retrieved). Each channel has its own counter, stored in a dual-port RAM. The refractory period is a specific duration of time during which the system ignores any potential action potentials that occur. This period is implemented to ensure that subsequent spikes are not erroneously detected in close temporal proximity to the initial spike. This design choice was made to effectively manage the available resources within the FPGA. By processing one channel at a time, the architecture optimizes resource utilization. Furthermore, this design choice led to the need to develop specifics blocks in Simulink from scratch. For instance, we had to design by hand the logic for the filtering, without exploiting ready to use blocks already designed in the libraries.

Although the testing was conducted on 32 channels, the architecture has the capacity to process up to 128 channels in real-time. Even if only a subset of the 128 channels is physically connected to the board, the architecture functions as if all channels are fully connected.

Data Encoding: after the processing stage, data encoding is employed to reconstruct the output data flow, ensuring that the transmitted data is in the appropriate format to be received and interpreted correctly by the Host PC GUI. To enable the storage of filtered data and timestamps of the identified spikes, the dataflow to the host PC was customized. While this customization provides the advantage of retaining this valuable information about the filtered data and spike timestamps, it came at the expense of the raw data.

By following this sequence of data decoding, processing, and encoding, the custom computation in Simulink can effectively transform the input data into a processed output that is appropriately formatted, ready to be transmitted to the host PC. It is important to highlight that each block, especially the signal processing block, has been designed to be as independent as possible from one another. This design approach allows for easy replacement of these blocks with user-defined blocks, providing flexibility and customization options. By designing the blocks to be modular and independent, the project enables users to replace or modify specific components according to their specific needs and requirements.

### D. FPGA Specifications

The Intan RHS is equipped with a Xilinx Spartan-6 FPGA. Specifically, the Xilinx XC6SLX45-2FGG484 FPGA with the following main features: i) 116 BRAMs of 18 Kb, ii) 58 DSP48A1 slices, iii) 43661 logic cells, iv) 6822 slices (containing 4 LUTs and 8 FFs), and v) 54576 FFs.

### E. Validation Dataset

The protocol for animal use was approved by the Italian Ministry of Health and Animal Care (authorization n. 509/2020-PR). The study included acute experiments conducted on 6 healthy adult Long-Evans rats (male, weighing between 300–400 g, aged 4–5 months) from Charles River Laboratories, Calco, LC. Upon performing neuroscientific experiments for another study [19], we then recorded spontaneous neural activity before the conclusion of the session. The experimental procedures were conducted at the Animal Facility of the Italian Institute of Technology (IIT), Genova, Italy.

**Fig. 4.**
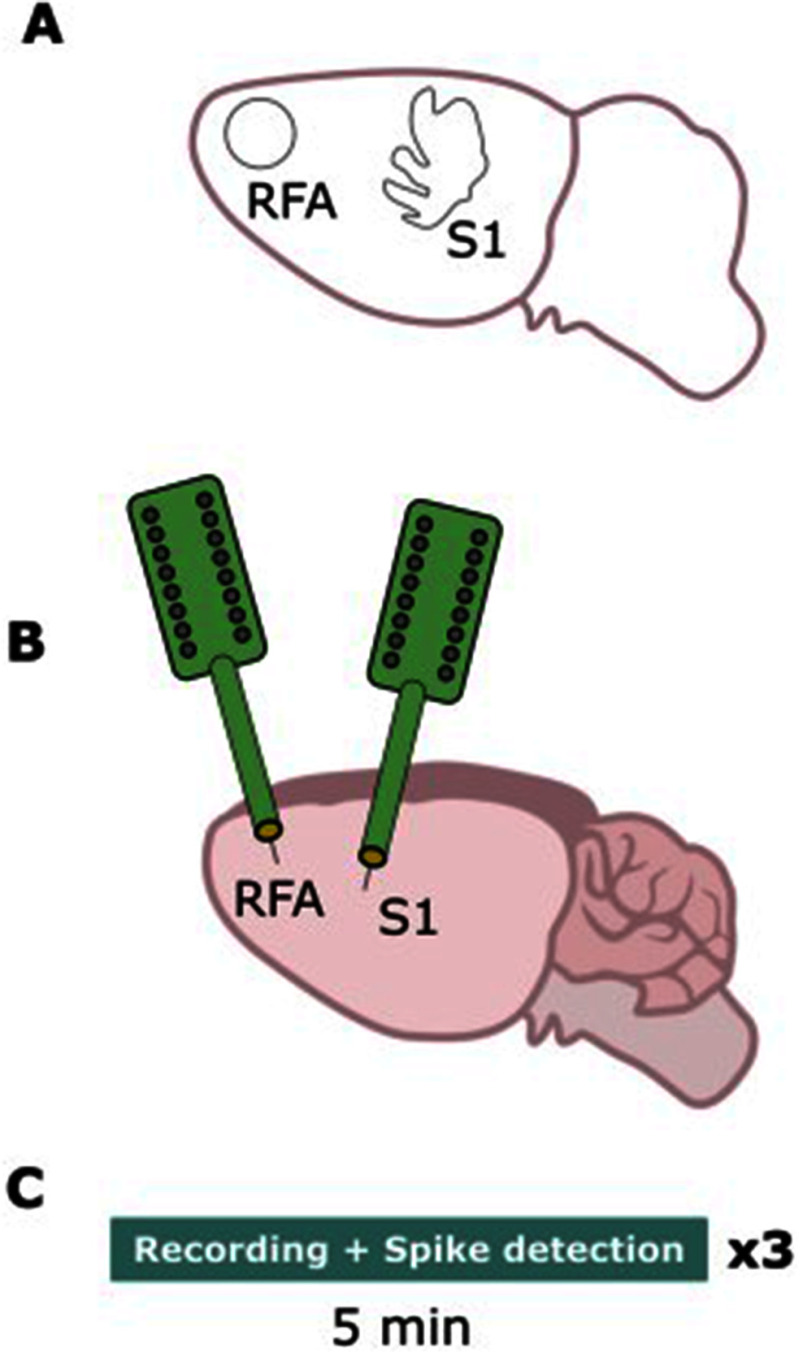
Experimental protocol. (a): Identification of the areas of interest by means of a stereotaxic frame. (b): placement of the electrode arrays in the rostral forelimb area (RFA) and in the primary somatosensory area (S1). (c): Timeline of experiments includes 5 minutes recordings and real-time spike detection from both areas per 3 times. Each time a different threshold has been used based on the experimental conditions (i.e., background noise level and spikes amplitude).

A 16-channel probe (NeuroNexus A4x4–5 mm–100–703–A16) was implanted in both RFA and S1 (see Fig. 4). The experimental protocol involved recording basal activity for 5 minutes from both electrodes. Real-time filtering and spike detection were performed enabling the CA (see Fig. 2). The extracellular signals were continuously sampled at a depth of 16 bits and a rate of 20 kHz or 30 kHz. For the spike detection algorithm, a refractory period of 1.5 ms was set and different thresholds ranging from −50 µV to −110 µV were configured based on the level of the background noise and the spikes amplitude. We chose thresholds as a multiplicative factor of the background noise as reported in literature [20]. For each animal 3 different thresholds [th1, th2, th3] have been set equal to [−50 µV, −70 µV, −90 µV] or [−70 µV, −90 µV, −110 µV].

## III. Results

### A. Hardware Utilization, Latency and Power Consumption

Resources utilization of the hardware is reported in Table I. It consists of the entire system resources usage: customized original Verilog code plus Verilog HDL code generated from Simulink.

**TABLE I:** FPGA Main Resources Usage. In Brackets the Percentage Usage With Respect to the Total Resources Available in the FPGA About: i) Flip-Flops (FFs), Lookup Tables (LUTs), Block RAMs (BRAMs), Inputs and Outputs (IOs), Digital Signal Processing Slices (DSPs) and Slices

FFs	LUTs	BRAMs	IOs	DSPs	Slices
8883 (16%)	17775 (65%)	69 (59%)	199 (62%)	8 (13%)	5927 (86%)

The system latency has been evaluated as the number of clock cycles required to detect a spike, factoring in the time to acquire the number of samples needed. Since the architecture is equipped with a 2nd order highpass filter, a minimum of two samples are required to perform the filtering, then the spike detection. Furthermore, the overall architecture can run at three different clock speeds: i) 84 MHz, ii) 70 MHz, and iii) 56 MHz, depending on the sampling frequency. This also depends on how the original architecture has been designed by the manufacturer. Consequently, we have obtained 66.24 µs, 80.29 µs and 100.37 µs of latency for sampling frequency of 30 kHz, 25 kHz and 20 kHz respectively.

Power consumption estimation has been performed via Xilinx XPower analyzer. The ambient temperature was set to 25 °C. On chip results shows 0.906 W of power consumption, requiring 1.248 W with a dynamic of 0.585 W for the power supply.

### B. System Validation

**Fig. 5.**
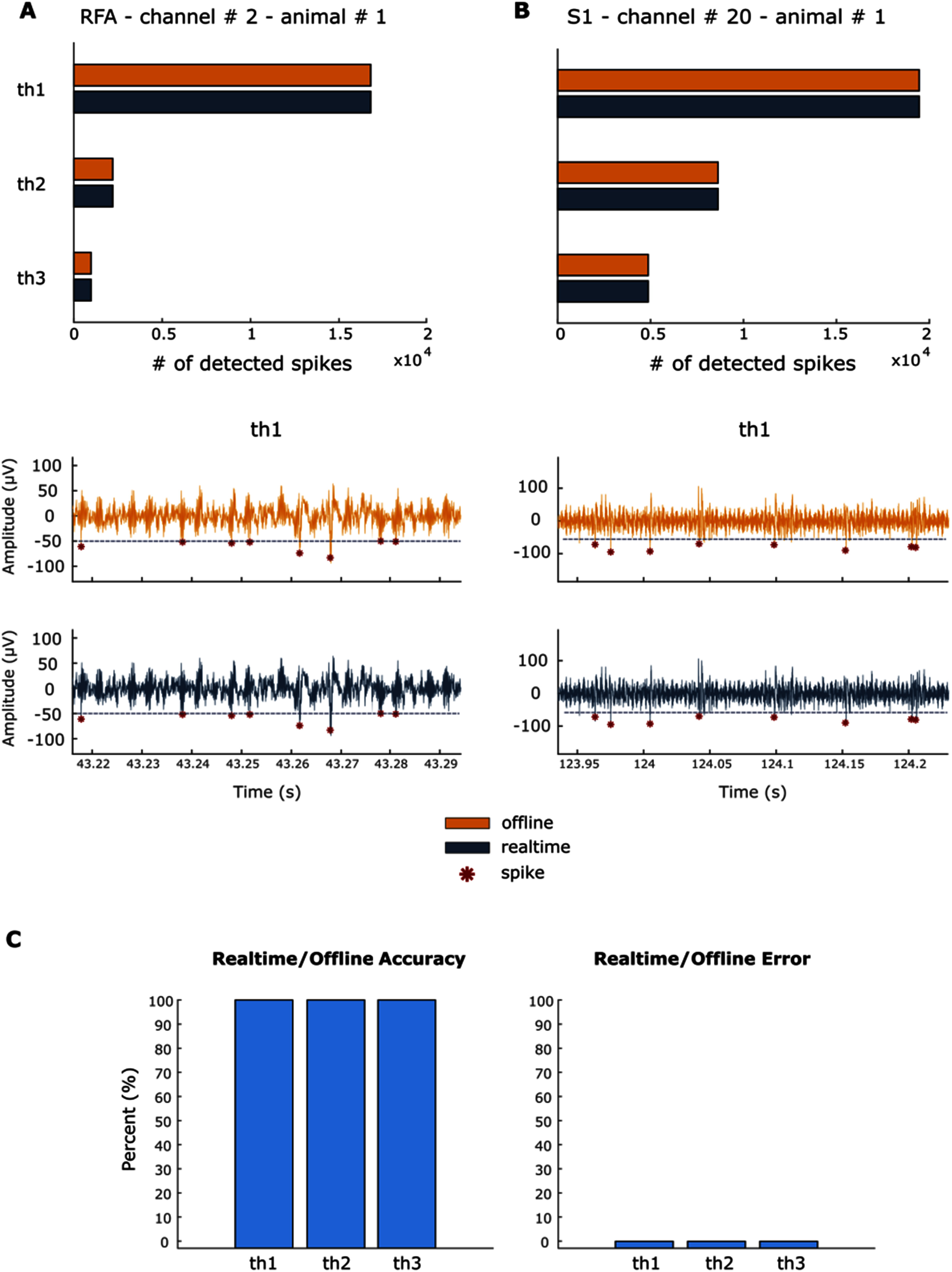
Panel A-B: Results for channel # 2 and channel # 20 of animal # 1. Panel C: Results overview. The orange color is referred to the offline output, while the blue color to the real-time one. A: number of detected spikes in RFA area for the 3 thresholds used (th1 = −50 µV, th2 = −70 µV and th3 = −90 µV). The number of spikes is always identical between offline and real-time implementation. Below, a zoom of the spike detected for the two implementations for th1. B: Number of detected spikes in S1 area for the 3 thresholds used (th1 = −50µV, th2 = −70 µV and th3 = −90 µV). The number of spikes is always identical between offline and real-time implementation. Below, a zoom of the spike detected for the two implementations for th1. C: These values have been obtained by averaging the accuracy and error values for each channel of each animal. On the left, the accuracy of the real-time over the offline is reported. A 100% accuracy is obtained in each case, showing that the real-time implementation behaves as the offline algorithm. On the right, the percentage error is shown. As acknowledged by the accuracy percentage, a 0% error has been obtained in each case.

A thorough comparison between offline and real-time implementation of the spike detection algorithms was conducted for each of the 32 channels across the 6 animals in the study. The offline hard threshold algorithm was applied to the dataset and the results compared to the output of the real-time implementation as shown in Fig. 5.

In Fig. 5 Panel A and B, exemplifying plots demonstrate the offline behavior versus the real-time behavior. For both areas, RFA and S1, the number of spikes identified by the implementation have been compared. In each case, the exact number of spikes and the exact same spikes in the signal are detected for the different thresholds.

To quantify the performance of the real-time spike detection, two metrics have been calculated: i) percentage accuracy and ii) percentage error. The percentage accuracy is defined as:

\begin{equation*}
{{E}_{acc}} = 100 - \left[ {\left( {\frac{{{{v}_o} - {{v}_e}}}{{{{v}_e}}}} \right) \cdot 100} \right] = 100 - \ \delta 
\end{equation*}

where ${{E}_{acc}}$ is the accuracy (or accuracy error), ${{v}_o}$ is the observed value (total number of spikes detected by the real-time algorithm), ${{v}_e}$ is the expected value (total number of spikes of offline algorithm) and δ is the percentage error. The percentage error is defined as:

\begin{equation*}
\delta = \left( {\frac{{{{v}_o} - {{v}_e}}}{{{{v}_e}}}} \right) \cdot 100
\end{equation*}

where δ is the percentage error, ${{v}_o}$ is the observed value and ${{v}_e}$ is the expected value.

The accuracy and error have been computed for each channel of each of the 6 animals and the overall average has been computed. As shown in Fig. 5 Panel C, we achieved 100% accuracy, then 0% error.

## IV. Discussion

By employing a Model-Based Design (MBD) methodology, we successfully implemented a state-of-the-art spike detection algorithm in real time that achieved accuracy equivalent to offline processing in demonstrator experiments. These experiments involved identifying neural events across two cortical regions in animal models in vivo. This accomplishment highlights the effectiveness of the MBD approach, showcasing its potential as a robust framework for designing and developing advanced neurotechnologies, even for non-expert hardware programmers.

Rather than designing a system from scratch, we adopted a strategic approach exploiting an existing commercial platform focusing on the development of custom computation modules inspired by a previous work of our group [21]. The MBD approach enables the development of complex architectures with a clear and structured design flow, offering several advantages over conventional methods ([22], [23], [24]), including a high level of abstraction, ease of implementation, and testing. This modular design philosophy enhances the processing pipeline to specific applications or research objectives, without extensive modifications of the entire system. Additionally, this approach fosters code reusability and collaboration within the research community, as custom-designed blocks can be shared and advanced further. To this end, our code is fully open access and available for any interested user.

In terms of architecture development, the decision to prioritize the storage of filtered data over raw data may restrict certain types of analysis. This limitation could be mitigated by adjusting the front-end architecture to accommodate the storage of both raw and filtered data. The choice of a hard threshold spike detection algorithm could be considered simplistic. However, it is widely used at the core of many spike sorting algorithms and closed-loop systems in neuroengineering [25], [26], [27]. Indeed, there is room for further improvement by implementing more advanced spike detection algorithms [17]. Notably, more sophisticated systems, such as those in [28], [29] have employed advanced spike detection techniques beyond what we have implemented here. However, none of these systems has incorporated the MBD methodology, which is the central focus of this manuscript. Another constraint of our system is the limited number of channels. Other acquisition systems allow recording from up to thousands of channels simultaneously [26], [29], [30], [31]. This constraint aligns with the capabilities of the technology used (i.e., Xilinx Spartan-6 FPGA), which also imposes limits on the complexity of algorithms that can be implemented in hardware, due to the residual resources. Indeed, such potential improvements must consider the computational complexity, memory requirements, and real-time processing constraints of the FPGA platform. Furthermore, with 65% of LUTs and 59% of BRAMs already in use, adding more complex algorithms may strain available resources, particularly if significant data storage and parallelization is required. Additionally, 86% of slices are utilized, but more advanced technologies could reduce resources usage and improve computational speed [28]. Lastly, although the system was validated on only 32 channels during in vivo experiments, the hardware design is capable of acquiring and processing data from up to 128 channels. The Simulink architecture is specifically optimized to utilize all 128 channels available in the Intan RHS system. Even if only a subset of channels is connected to the board, the architecture functions as if all channels are active.

Here we emphasize that the potential of an MBD approach lies in its ability to significantly reduce development time. As previously demonstrated in [32], we also found that MBD leads to a quicker generation of HDL descriptions compared to manual methods. Additionally, Simulink provides a more intuitive and faster learning experience than traditional HDLs like VHDL or Verilog. This streamlined approach allows researchers to focus on algorithm design rather than the complexities of hardware design. While Simulink's graphical interface may limit fine-grained control in hardware design, it excels in facilitating rapid prototyping and system-level modeling. This enables researchers to efficiently explore and iterate algorithmic concepts and system architectures, making the development process more efficient and accessible.

## V. Conclusion

In conclusion, the MBD approach described in this work represents a significant contribution to the development of technologies for neuroengineering and neuroscience. This project is actively being developed and is available to the community in open access modality. As part of this ongoing development, new features and enhancements will be introduced over time to expand and improve the current capabilities, by e.g., adding new detection algorithms. Making the project available to the community not only encourages collaboration and knowledge sharing but also opens up opportunities for contributions from the wider user base. This community-driven approach will enable the system to evolve, in line with the needs and requirements of its users.

## Supplementary Materials

You are encouraged to visit the supplementary materials document to deepen your understanding about: (i) FPGA architecture of Intan RHS system, (ii) how the Simulink model is integrated in the original Verilog code of the Intan RHS system, (iii) graphical user interface description and changes provided to enable custom architecture from a host PC, (iv) Simulink library developed to allow for Simulink model customization, and (v) surgical procedure details.

Supplementary Materials
