# Supplementary material for: Enabling Model-Based Design for Real-Time Spike Detection
Source: IEEE Open J Eng Med Biol. 2025 Feb 3;6:312–9. doi: 10.1109/OJEMB.2025.3537768 (PMC12250937; doi:10.1109/OJEMB.2025.3537768)
Supplement: Supplementary Materials [file supp1-3537768.pdf]

Supplementary Materials

Enabling Model-Based Design for Real-Time Spike Detection

M. Di Florio, Y. Bornat, M. Carè, V. R. Cota, S. Buccelli, M. Chiappalone*

I. Intan RHS System FPGA Architecture Overview

To better understand the Simulink® custom architecture (CA) architecture, it's important to have a brief overview of the original architecture of the Intan RHS system.

The Intan RHS Stim/Recording controller is an open-source commercial device that enables recording and stimulation from 128 electrodes at the same time. The system's core is the Opal Kelly XEM6010-LX45, a USB 2.0 integration module based on the Xilinx Spartan-6 FPGA. Opal Kelly provides a FrontPanel SDK for configuring communication and interfacing the FPGA with a PC, Mac, or Linux hardware, reducing the effort required to build functional prototypes.

Intan Technologies offers a host computer application programming interface (API), written in C++, for multi-platform support (Qt), which handles communication with the FPGA and displays the graphical user interface (GUI). Additionally, they provide hardware description language (HDL) code, written in Verilog, that manages real-time communication with the RHS2116 digital electrophysiology stimulation/amplifier chips and the host computer. The RhythmStim code, developed for the Opal Kelly board, enables streaming of up to 128 amplifier channels from multiple RHS2116 chips, data from up to 8 other ADCs, and signals from up to 16 digital inputs. It allows for setting stimulation protocols for all 128 channels, with settings such as amplitude, pulse width, and sequences configurable through the GUI. The data is synchronized, time stamped, and transmitted over a standard USB 2.0 bus to the host computer at a rate exceeding 20 MByte/s. The core of the RhythmStim code is a Finite State Machine (FSM) that cycles through 140 repeating states to execute a single serial peripheral interface (SPI) cycle. Each SPI cycle involves executing a command like "convert(0)" to retrieve the actual value of channel "0" of all the connected headstages, and transferring the data to the FIFO within the same 140 states. A counter is then incremented from 0 to 19 to track the series of 20 commands sent to the RHS2116 (16 "convert" commands for each amplifier channel and 4 auxiliary commands for enabling stimulation channels and other operations). This FSM is the principal component of the overall original architecture, allowing to handle the sampled data from the headstage redirecting them to the Host PC. By intercepting this stream of data, we were able to process the samples in real-time.

II. Custom Architecture I/O Interface

To guarantee communication between the original and custom paths, an input/output interface has been designed using Verilog (see Fig 2 Panel A2 of the main manuscript).

List of input ports:

- FIFO_data_in: this input represents the recorded data from the headstage;
- FIFO_write_to: this input is a flag that indicates the validity of the retrieved data. If the value is 1, the data retrieved is informative. Otherwise, it is 0;
- data_stream_en: this is an 8-bit register that provides information about the enabled headstages and the port they are connected to on the controller. A value of 1 in position 0 indicates that the first headstage is connected. A value of 1 in position 0 and in position 1 indicates that the first and second headstages are connected and will send values;
- pipe_in_data: this input contains the pipe-in endpoint values that come from the Qt GUI. This port is useful for sending custom parameters from the host PC;
- pipe_in_en: this input contains the pipe-in enable endpoints that come from the Qt GUI. If the value is 1, it means that a command was sent through pipe-in data. This port informs the FPGA about the validity of the data sent from the host PC;
- pipe_out_read: this input is currently unused and left for future development;
- cfg: this input is left for future development and is intended to inform the custom architecture about the current configuration version.

List of output ports:

- comp_data_out: this port contains the recorded data to be transmitted to the Host computer via the USB interface;
- comp_data_out_valid: this boolean port becomes true when comp_data_out contains valid data to be transmitted via the USB interface;
- trig_stim: this port is reserved for future development and will send triggers for stimulation to close the loop;
- computing: this port is designed to inform external modules that the custom architecture is still computing and not yet ready to process other tasks. It is currently not in use;
- pipe_out_data: this endpoint is intended for debugging. It is useful for sending informative data from the FPGA to the Host PC;
- status: this port will indicate the status of the custom architecture module.

In summary, the signal dataflow is redirected through the custom path where real-time processing takes place. This custom path is built using Verilog code generated by a

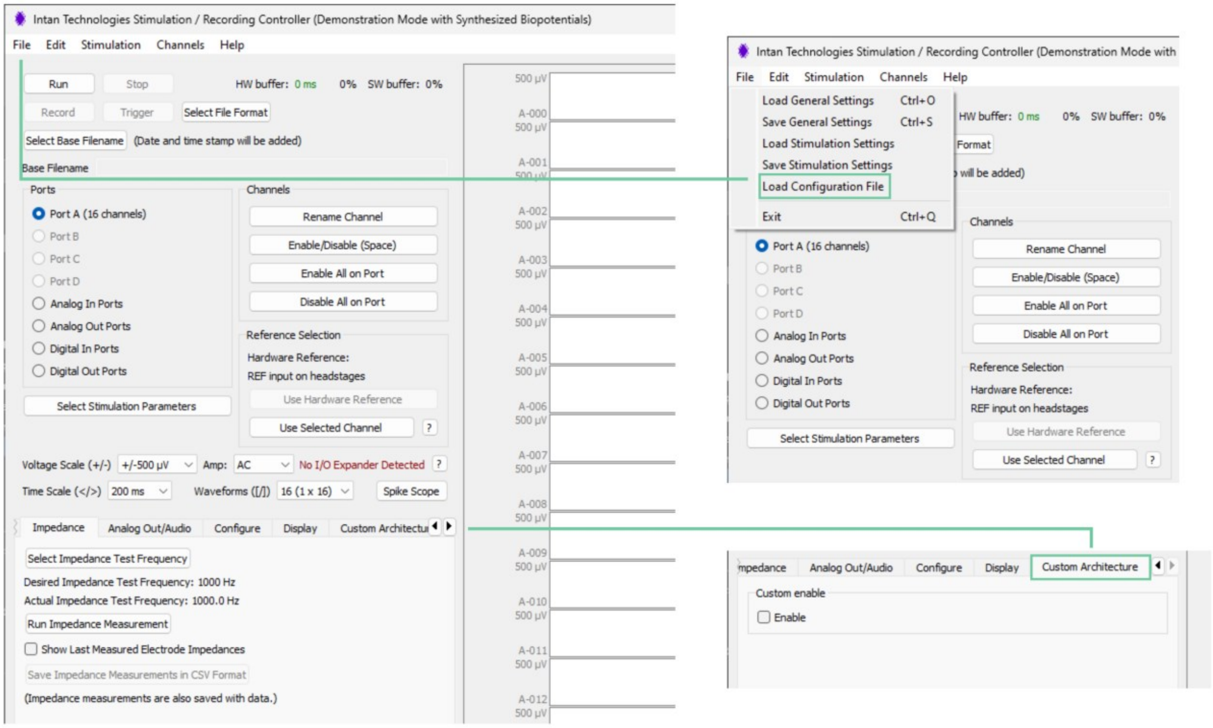

Fig. 6. Customized GUI. An item called "Load Configuration File" in the File windows has been added to allow for the loading of a configuration file which includes the threshold value and the refractory period to apply. A custom tab called "Custom Architecture" has been added with a flag to enable the dataflow redirection to the custom path. If this flag is not enabled, the system maintains its original behavior.

Simulink® model, allowing users to customize the Verilog architecture and implement their own algorithms. At present, custom processing involves a high-pass filter and hard threshold spike detection which looks for a local peak in the signal.

III. Graphical User Interface Customization

Overall, the GUI allows for real-time multichannel data visualization and customization of all registers of the headstage (i.e., RHS 2116) in order to control the acquisition/stimulation loops. The user can set the sampling frequency, load default settings file, store the data, perform some simple signal manipulation, choose from which channel delivers stimulation, communicate with external devices via analog/digital IOs, etc.To enable the rerouting of the dataflow through the custom path, and the delivery of custom parameters to the FPGA, the manufacturer's original open-source C/C++ code has been modified (see Figure 6). These modifications allow for the integration of the custom computation within the existing codebase. The original graphical user interface (GUI), which was developed using Qt 5.8, serves as the front-end for the USB interface of the Opal Kelly XEM6010-LX45. The GUI has been extended to include a new tab called "Custom_arch." Within this tab, there is a flag named "Cst_en" that can be toggled to activate the custom computation. When the "Cst_en" flag is set, a specific register of the architecture is written, triggering the switch to the custom architecture. This mechanism ensures that the dataflow is redirected through the custom computation path when the flag is enabled (see Fig. 2 Panel A1 of the main manuscript). In the File windows of the system's GUI, a new item called "Load Configuration File" has been added. This functionality allows users to specify custom threshold values and refractory periods for the spike detection computation. By selecting this option, users can load a text file organized in a specific format. The text file must contain the customized threshold and refractory period values for the spike detection algorithm. It is structured in a way that facilitates easy loading of the custom parameters. The txt file is read, and the content stored in an array. The array in then sent to the FPGA via USB. At hardware level, a specific state machine interprets the array and updates the values of the registers for the thresholds and refractory period. At the current state of the project, the same threshold and refractory period values are applied to all channels. This feature provides flexibility for users to adjust the spike detection parameters according to their experimental conditions. The software, including the modified GUI and the custom computation code, has been compiled using Qt Creator community edition. It has been specifically compiled for the Windows 10 64-bit operating system, ensuring compatibility with the target environment. These modifications and adaptations enable the integration of the custom computation within the existing software infrastructure, allowing for the seamless execution and control of the custom architecture through the modified GUI.

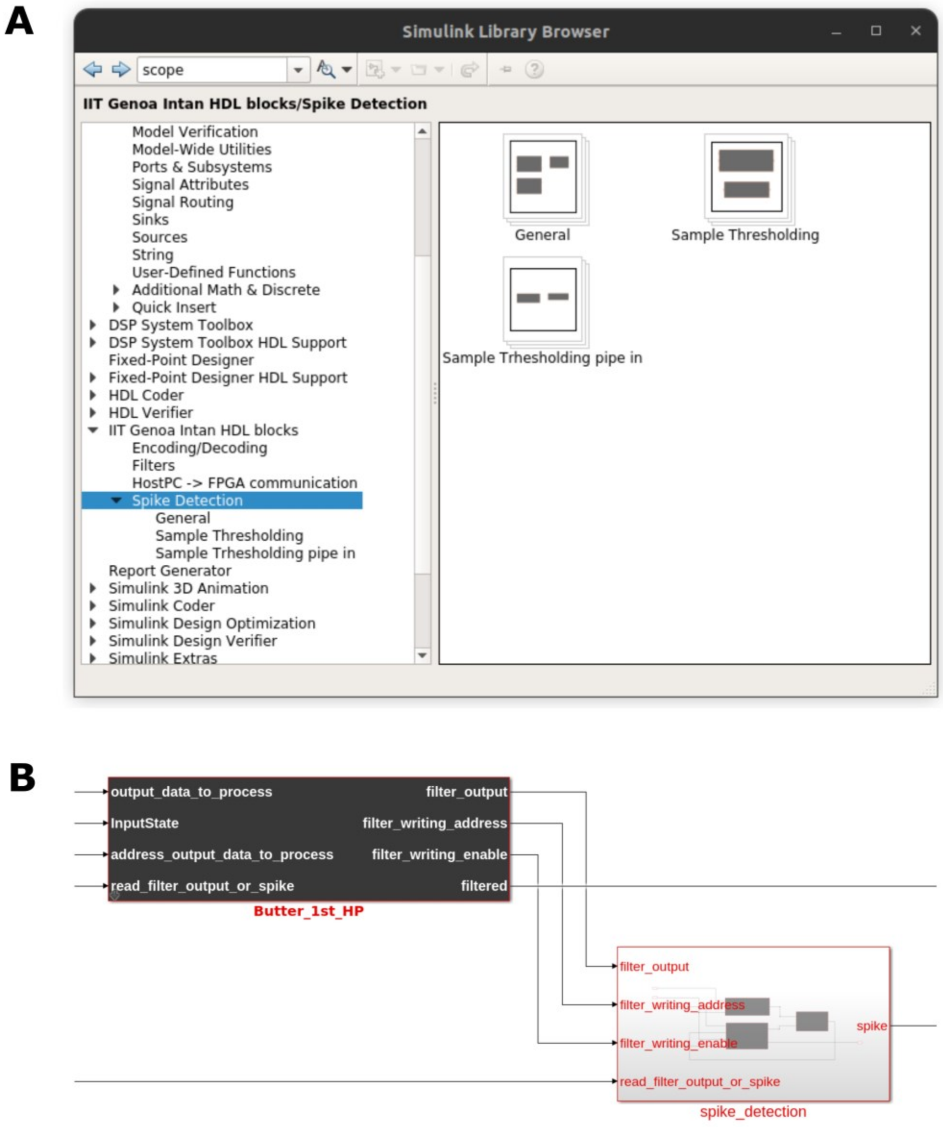

Fig. 7. Simulink® library. A: library developed for Simulink®. It can be installed once downloaded the GitHub repository. It provides specific block for the design of spike detection algorithms already tested and validated for the Intan RHS. B: Example of application. A user can drag and drop its desired blocks into the Simulink® environment and, following the name of the I/O ports, he can intuitively connect them.

IV. Simulink® Library

A custom Simulink library has been developed (see Fig. 7). This library contains preconfigured blocks that are specifically designed to interface with the Intan RHS system, simplifying the development process. The custom Simulink library includes a collection of blocks that have been preconfigured with the necessary parameters and settings to seamlessly integrate with the Intan RHS system. These blocks can be easily connected and configured within the Simulink environment, allowing users to design and customize their own algorithms without requiring specialized knowledge in FPGA programming or hardware design.

V. Surgical Procedures for Data Acquisition

Rats were anesthetized by placing them inside a vaporizing chamber and injecting gaseous isoflurane (5% @ 1 lpm). To achieve surgical level anesthesia, ketamine (80-100 mg/kg IP) and xylazine (5-10 mg/kg IM) were administered. The rat was then secured in a stereotaxic frame, and vital parameters were monitored throughout the procedure. A midline skin incision was made after applying lidocaine cream as a topical analgesic. A laminectomy was performed successfully at the level of the Cisterna Magna to allow the cerebrospinal fluid (CSF) to drain. Burr holes (3 mm diameter) were made over the primary somatosensory area (S1) and rostral forelimb area (RFA) based on stereotaxic measurements [9] at –1.25, +4.25 and +3.5, +2.5 AP, ML, respectively. Finally, the dura mater was removed

from both burr holes to allow insertion of MEAs (Neural Probes; A4x4-5 mm-100-125-703-A16, NeuroNexus).
